# HumanBrainAtlas: an in vivo MRI dataset for detailed segmentations

**DOI:** 10.1007/s00429-023-02653-8

**Published:** 2023-06-05

**Authors:** Mark M. Schira, Zoey J. Isherwood, Mustafa S. Kassem, Markus Barth, Thomas B. Shaw, Michelle M. Roberts, George Paxinos

**Affiliations:** 1 School of Psychology, University of Wollongong, Wollongong, NSW 2522 Australia; 2 Neuroscience Research Australia, Randwick, NSW 2031 Australia; 3 Department of Psychology, University of Nevada, Reno, NV 89557 USA; 4 Centre for Advanced Imaging, The University of Queensland, St Lucia, QLD 4067 Australia; 5 School of Information Technology and Electrical Engineering, The University of Queensland, Brisbane, QLD 7067 Australia; 6 School of Psychology, The University of New South Wales, Sydney, NSW 2052 Australia

**Keywords:** MRI, Human brain, 7T, 3T, T1w, T2w, DWI, Image acquisition

## Abstract

We introduce HumanBrainAtlas, an initiative to construct a highly detailed, open-access atlas of the living human brain that combines high-resolution in vivo MR imaging and detailed segmentations previously possible only in histological preparations. Here, we present and evaluate the first step of this initiative: a comprehensive dataset of two healthy male volunteers reconstructed to a 0.25 mm isotropic resolution for T1w, T2w, and DWI contrasts. Multiple high-resolution acquisitions were collected for each contrast and each participant, followed by averaging using symmetric group-wise normalisation (Advanced Normalisation Tools). The resulting image quality permits structural parcellations rivalling histology-based atlases, while maintaining the advantages of in vivo MRI. For example, components of the thalamus, hypothalamus, and hippocampus are often impossible to identify using standard MRI protocols—can be identified within the present data. Our data are virtually distortion free, fully 3D, and compatible with the existing in vivo Neuroimaging analysis tools. The dataset is suitable for teaching and is publicly available via our website (hba.neura.edu.au), which also provides data processing scripts. Instead of focusing on coordinates in an averaged brain space, our approach focuses on providing an example segmentation at great detail in the high-quality individual brain. This serves as an illustration on what features contrasts and relations can be used to interpret MRI datasets, in research, clinical, and education settings.

**Supplementary Information:**

The online version contains supplementary material available at 10.1007/s00429-023-02653-8.

## Introduction

The investigation of human brain anatomy has a long history, and for most of this time has exclusively relied on the study of post-mortem brains. Today, clinicians and many neuroscientists use Magnetic Resonance Imaging (MRI) to acquire images from living human brains, hence the need to identify brain anatomy in these images. There are a number of projects aiming to provide assistance with brain anatomy, such as the BRAIN Initiative (Underwood 2013), the Human Connectome Project (HCP) (Van Essen et al. 2013), the Big Brain (Amunts et al. 2013; Amunts and Zilles 2015), Brainnetome (Jiang 2013), the scalable Brain Atlas (Bakker et al. 2015), and the Allen Brain Atlas (Hawrylycz et al. 2012; Sunkin et al. 2013). While these approaches are powerful, there are still major needs faced by contemporary clinicians and researchers not addressed, principally anatomical resolution. For example, population-bases atlases, by their nature, average out and warp many MR images to a best fit, resulting in loss of detail. As a result, population-based atlases of the human brain typically identify about 50 structures, a handicap for those interested in identifying small structures in an individual brain. Histology-based atlases are more comprehensive, identifying as many as 800 structures (Mai et al. 2016a), but histology is limited—by its very nature, it is post-mortem and the tissue appearance is significantly different from in vivo MR images.

Technological advances have progressively improved the quality and spatial resolution of MRI, including the use of specifically tailored MRI acquisition techniques (MR sequences and protocols), e.g., 3D acquisitions using small, isotropic voxels (Suddarth and Johnson 1991) which was enabled by the use of higher magnetic field strength (Budde et al. 2014; Pohmann et al. 2016; Ugurbil 2012) and multi-channel array coils (Roemer et al. 1990)—all measures that improve the signal-to-noise ratio (SNR) dramatically. MRI techniques permit acquisition protocols that provide satisfactory anatomical detail from in vivo scans of control subjects, using widely available clinical hardware (Busse et al. 2006; Marques et al. 2010). Advancements now allow levels of resolution that have been previously thought unattainable––recent work demonstrating a high degree of structural information by combining the right acquisition protocols with suitable image processing (Avants et al. 2010, 2011; Janke et al. 2016; Lusebrink et al. 2021). Despite this progress, anatomical delineations of small structures are often inadequate in both cortex and subcortex, with many delineations restricted to gross neuroanatomy, or when detailed they are parochial, covering only segments of the brain (Iglesias et al. 2018; Sone et al. 2016; Winterburn et al. 2013).

While efforts have been made to develop methods for reliable atlas segmentations using minimal, or AI-assisted user input (Diaz-Pinto et al. 2022; Luo et al. 2021; Zhang et al. 2021), time-intensive manual segmentations on high-quality templates are still considered by many to be the ‘Gold Standard’ for downstream medical imaging processing (Bauer et al. 2013). Atlases not only provide spatial prior probabilities for many segmentation algorithms, but also a reference point for clinical research studies (Ashburner and Friston 2009; Ashburner et al. 1998; Avants et al. 2010; Awate et al. 2006; Eickhoff et al. 2018; Van Leemput et al. 2003; Wang et al. 2013). When aligning to stereotaxic space, the choice of atlas is often driven by use-case similarities to contrast, field strength, or population characteristics. While there is a push to generate appropriate matching conditional atlases to user input via (for example) machine learning (Balakrishnan et al. 2019; Dalca et al. 2019a, b; Hoffmann et al. 2021; Hoopes et al. 2021) or through larger multi-site cohort studies (Fillmore et al. 2015; Richards et al. 2016), there is still a great need for *accurate delineations* of anatomical landmarks in in vivo atlases that are of high quality and of similar shape and intensity characteristics as in vivo data inputs.

In summary, there is a need for a new, comprehensive, and stereotaxically accurate map of the human brain for in vivo neuroimaging applications. The HumanBrainAtlas (HBA) addresses these limitations, combining the anatomical resolution of histology with in vivo MRI to remove handicaps each technique possesses. In doing so, it will elevate the detail of MRI segmentations to the level of histology. It closes the gap between existing population-based efforts and histology by focusing on the individual instead of the population. Leveraging high-quality individual data also shifts the primary aim. While population-based atlases provide a set of coordinates indicating the likely position of structures in some standard space, our approach provides a detailed reference on the anatomical organisation. It provides relative locations shapes and layouts of structures; it illustrates structure contrasts in in vivo MR images, thereby linking post-mortem techniques to in vivo MRI.

To this end, HBA renders two living subjects in a ultra-high-resolution MRI of 250 microns voxel grid. As in the histological atlas of Mai et al. (2016a, b), we aim to define approximately 800 structures, providing similar accuracy for science and clinical practice, but within the much more ubiquitous and clinically relevant space of in vivo MRI. Our ambition is to link the field of post-mortem anatomy to in vivo MRI. For this goal, we present herein high-resolution MRI data at 7 T (T1w and T2w) and 3 T (DWI). The general approach was to collect repeated images at maximum permissible resolution and average these individual, grainy images at even greater resolution to construct a super-resolution average of individual brains, bringing the neuroanatomical resolution of histology to the world of MRI. The datasets, post-processing protocols, and ongoing progress of delineations are made available for open access through our website hba.neura.edu.au and https://osf.io/ckh5t/.

The aim of this manuscript is to introduce the resource, how it was constructed and to demonstrate the utility of the data acquired for the purpose to support delineation rivalling the detail of histological atlases.

## Methods

### Subjects

Two male subjects were scanned extensively (up to 20 sessions) for this project. Both were healthy and with no history of neurological or psychiatric conditions. While some scanning parameters differed between subjects, for the most part, each subject underwent a similar set of scanning protocols. At the time of scanning, Subject 1 was 45 and Subject 2 was 30 years old.

### Scanning acquisition parameters

T1w, T2w, and Proton Density (PD) images were acquired on a Siemens 7 T MAGNETOM at the Centre for Advanced Imaging using a 32-channel head coil across multiple sessions (up to 12 per subject). Diffusion Weighted Imaging (DWI) data were acquired on a Philips 3 T Ingenia CX at the NeuRA Imaging Centre using a 32-channel head coil, again across multiple sessions (up to 10 per subject). More specific details for each protocol are listed below.

### T1w

Three different T1w protocols were used, here called MP2RAGE, Dutch and FLAWS. All three are based on the Siemens WIP 944 (a two-inversion MP2RAGE sequence), but using different parameters. The idea being that each sequence will be advantageous for slightly different regions, and the resulting average will benefit from each. The exact scan protocols can be found in the supplementary materials. Briefly, the parameters are: (a) MP2RAGE Protocol: Voxel size 0.4 mm isotropic, TR = 4300 ms, TE = 1.8 ms, TI1 = 700 ms, TI2 = 2370 ms, FA1 = 4˚, FA2 = 5˚, GRAPPA = 2, Echo spacing 5.4 ms, Bandwidth 590 Hz/Px, and the denoised (UNIDEN) image was used; (b) Dutch Protocol (Fracasso et al. 2016): Voxel size = 0.5 mm isotropic, TR = 6000 ms, TE = 3.18 ms, TI1 = 1200 ms, TI2 = 4790 ms, GRAPPA = 3, FA1 = 8˚, FA2 = 9˚, Bandwidth = 630 Hz/Px, the PD corrected Inv1 image was used; and (c) FLAWS Protocol: Voxel size 0.6 mm isotropic, TR = 5000 ms, TE = 1.49 ms, TI1 = 620 ms, TI2 = 1450 ms, FA1 = 4˚, FA2 = 8˚,GRAPPA = 3 partial Fourier 6/8 Bandwidth = 630 Hz/Px, and the PD corrected Inv2 image was used.

We trialled a number of different scanning protocols, continuously optimising them. For some trialled sequences, the results were not used for the averaged datasets presented here. For other sequences, their data were included into the averages, even as they proved somewhat less effective than other sequences. Specifically, the sequence called MP2RAGE revealed the most detail and structure (see Fig. S6). Nevertheless, the data provided from the Dutch and FLAWS scans were integrated and improved the T1w average.

### T2w

T2w images were collected with a 3D TSE sequence (SPACE) using the Siemens WIP692; again, detailed parameters can be found in the supplementary materials. Briefly, TR = 1330 ms, TE = 118 ms, GRAPPA 3, SPAIR fat suppression, 384 slices, FOV 256 × 256 × 154 mm, Matrix size 640 × 640, resolution 0.4 mm isotropic, Bandwidth = 521 Hz/Px.

### Proton density (PD)

In each session, one proton density (PD) scan was collected, Voxel size 1 mm isotropic, TR = 6.0 ms, TE = 3.0 ms, GRAPPA = 2. This PD was used to correct for intensity inhomogeneities present in T1w scans for the same sessions (Van de Moortele et al 2009).

### Diffusion-weighted imaging (DWI)

DWI data were acquired on a 3 T Phillips Achieva CX, at the NeuRA Imaging Facility in Randwick, Australia using a diffusion weighted (DTW) echo planar imaging (EPI) sequence. Native scan resolution was 1.25 mm isotropic, field of view (FOV) 240 × 200 × 147.5 mm, 118 slices, 32 directions, 4 b-factor averages, B-val = 1000, TE = 60 ms, TR = 26.5 s, SENSE = 3, SPIR (Spectral Saturation with Inversion Recovery) fat saturation. Fat shift direction was A to P, inverse blip scans were collected for distortion correction and the total scan time was 52 min. Ten scans were acquired with one scan per session to ensure maximum subject compliance with minimal motion.

### Data analysis

#### T1w and T2w pre-template preprocessing

Each T1w scan type (MP2RAGE, Dutch, Flaws) was preprocessed independently through the following steps.

1. Applying the ImageMath command from the ANTs toolbox to truncate the luminance intensities of each scan with 0 as the lower quantile and 0.999 as the upper quantile.
2. Using the robustfov script from FSL to reduce file size by removing unnecessary parts of the scan (neck, nose, etc.).
3. Upsampling (b-spline interpolation) the voxel size to 0.25 mm isotropic to ensure that voxel sizes were uniform across different scan protocols and modalities. This decreases the effect of blurring caused by ‘reslicing’ or ‘resampling’ and allows some degree of super-resolution by integrating information over multiple frames (Farsiu et al. 2004; Manjón et al. 2010; Tsai 1984; Van Reeth et al. 2012) to increase detail. A voxel resolution of 0.25 mm also provides the benefit of allowing extraction of slice images at 0.5 mm interval for the segmentation process without any additional resampling.
4. Skull stripping was undertaken to improve alignment by removing parts of each scan that did not include cortex. We also conducted skull stripping due to the large file size of our raw scans (~ 1–2 Gb), because skull stripping decreased file size considerably (~ 300 Mb), by removing noise outside of the brain. For this, we used HD-BET to create a brain mask for each scan (Isensee et al. 2019). To avoid this mask removing brain areas with low signal (e.g., temporal cortex), we also created a skull stripped variant with the brain mask inflated by 3 mm. Accurate skull stripping is critical and results were carefully inspected. When the skull strip was inaccurate for a specific scan, an accurate skull strip mask from another dataset was used. For this, the two datasets were aligned, and when this alignment was successful, the accurate mask was transformed. Remaining inaccuracies in skull stripping were manually corrected using the ITKSnap software.
5. Ensuring all the dimensions of each scan was 1024. As in Step 3, this was used to ensure uniform dimensions across different scan protocols and modalities. With background being zero values, this did not increase file size because the files were saved in a zipped format (nii.gz).
6. Proton density correction: We used the method proposed by Van de Moortele et al. (2009) to correct for luminance inhomogeneities in our T1w images. Proton density images were collected during the same scan session for each T1w image, and the T1w images acquired using the ‘Dutch’ and the ‘FLAWS’ protocol were divided by the aligned Proton Density image. This yielded images with much reduced inhomogeneities and high grey/white contrast.
7. All T1w and all T2w images were then aligned using FLIRT and a linear average was generated using the fslmerge command with the –t flag and then averaged using fslmaths. This created one unbiased linear average for each for T1w and T2w set of scans, avoiding influence by the order in which the scans were collected. This linear average was used as a starting point for symmetric group-wise template generation.

#### Template generation

Symmetric group-wise normalisation was conducted using Advanced Normalisation Tools (ANTs), specifically using the antsMultivariateTemplateConstruction.sh script. This employed a cross correlation similarity metric and a Greedy SyN transformation model for non-linear registration. We used 20 × 15 × 5 as the maximum number of steps in each registration, the gradient was 0.1, and the total number of iterations was 3. These parameter settings were based of the settings used by Lüsebrink et al. (2017).

Dutch, FLAWS, and MP2RAGE were all integrated into a single fit for a T1w template, but each modality (T1w, T2w, and DWI) was processed separately and aligned afterwards using ANTs non-linear registration. A non-linear alignment was chosen after the results of affine alignments between templates were found to be good, but still marginally suboptimal. This was specifically noticeable in the computed ColorT1T2, which we observed to be sharper after non-linear alignment. Supplementary Figures S1 and S2 show a simplified flowchart of the data processing pipeline. For comparing the different T1w scan protocols, we fit additional models for each scan type. These are compared in Supplementary Fig. S7.

### Diffusion-weighted imaging (DWI)

DWI data were analyzed using MRtrix 3.0.2 (Tournier et al. 2019) and ANTs. First, each scan was up-sampled using sinc interpolation by a factor of 2 × 2 in the inplane direction (to 0.625 mm × 0.625 mm × 1.25 mm) and then preprocessed using the dwifslpreproc script providing top-up distortion correction and eddy_cuda correction (Andersson and Sotiropoulos 2016; Jenkinson et al. 2012) with the eddy options “–slm = linear”. Then, the preprocessed data were upscaled across slices, again by a factor of 2, so the final output had an isotropic resolution of 0.625 mm. This upsampling strategy was chosen to increase the spatial detail through averaging repeated acquisitions. Doubling the across slice resolution is incompatible with eddy correction, and hence, only the inplane direction can be up-sampled before eddy correction. For each of the ten up-sampled preprocessed DWI datasets, a mean DWI image, an FAC (Fractional Anisotropy Colour) image, as well as an FOD (Fibre Orientation Distribution, dwi2fod) using dhollander algorithm (Tournier et al. 2019) were calculated.

Subsequently, the ten mean DWI images were aligned using the ANTs MultivariateTemplateConstruction.sh script, with a template resolution of 0.5 mm. This resulted in a high-resolution high-quality mean DWI image, essentially displaying a T2*w contrast with good image contrast and detail. The transforms estimated from this were then applied to the 10 FAC images and the 10 FOD images (via 10 ID images) using the mrtransform function from MRtrix. This ensured that the FOD vectors were transformed correctly (using the option -reorient_fod yes). FAC images and FOD were then averaged in the DWI template space using mrcalc. This intermediate 0.5 mm space was used to save RAM and compute resources, but being compatible to the 0.25 mm overall template. Also averaging 10 FOD images at 0.25 mm would have required above 128 GB of RAM. Then, all DWI data were transformed into the final template space (0.25 mm) using ANTs for the FAC and mean image and ID files and mrtransform for the FOD. Finally, from the average FOD, a DEC (direction encoded colour) image was calculated using the T1w template for panchromatic sharpening (fod2dec -contrast, Dhollander et al. 2015).

### Template alignments, multi-contrast images, and final contrasts

Great care was taken to achieve optimal alignment of these different image modalities, overcoming the slight distortions in each. We computed multi-contrast images, to validate the accuracy of these alignments, where alignment inaccuracies would introduce blur into these multi-contrast images. First, the image “ColorT1T2” was calculated from the T1w and T2w by manipulating the RGB (red, green, blue) channels of the image (Table 1). The red channel of the image was calculated as T1w/T2w (using Matlab). This was thresholded at the 96th percentile, because the division resulted in some extremely large values in the background. The green channel of the image is the T1w and the blue channel is the T2w image. This colour scheme was chosen, because it highlights some blood vessels in red, greatly simplifying the discrimination of blood vessels and nerve fibres in the manual segmentation.

The second multi-contrast image is called DEC_T1 and it combines the T1w and DWI data. Specifically, it is the output of the fod2dec function encoded image derived from the FOD.

**Table 1:** Summary of the MRI scans included in the HumanBrainAtlas project

Name	Derived from scans	Description
T1w	7 T MP2RAGE, DUTCH, FLAWS	T1-weighted scan. The main reference contrast, derived by averaging all T1w scans of sufficient quality
T2w	7 T 3D TSE sequence (SPACE)	T2-weighted scans
DWI_average	3 T SPIR EPI	Average of the distortion-corrected EPI images, mostly used for aligning the DWI data to the T2w (and hence the T1w) image
FAC	3 T SPIR EPI	For every DWI scan, an FAC image was calculated. These ten FACs were aligned with the T2w and averaged
DEC_T1	T1w, 3 T SPIR EPI	Direction-encoded fractional anisotropy reconstructed from the FOD (fod2dec -contrast)
ColorT1T2	T1w and T2w	A derived image combining the T1w and T2w contrast—helpful for segmentation and highlighting of blood vessels

### Detailed documentation of the processing scripts

We provide a detailed “How to” like description of the data processing online.

1. WITHOUT an input template: https://hackmd.io/DQbUc5lJQ_u9qCyd3Q3kSA
2. WITH an input template: https://hackmd.io/qKZtbfpSTnGzSebiQW3KfQ
3. Using SLURM to run ANTs on the ‘Massive’ platform (massive.org.au): https://hackmd.io/b5YqltBPRgqxXl83ZVbjcQ
4. DWI data: https://hackmd.io/0W2D1db_QROG_HU9Vv4-JA

### Delineation of neuroanatomy

For each 0.5 mm, a set of three orthogonal planes were extracted: coronal, sagittal, and axial (horizontal). For each plane, a series of images were compiled per voxel step, from each of their respected ranges; i.e., for coronal, from 65 mm before the anterior commissure (− 65AC) to 80 mm after the anterior commissure (+ 80AC). These MR slice images are analogous to histological sections, allowing direct and practical comparisons with existing delineated histology slices.

With these series of images, across all contrasts, a set of four ‘virtual’ fiduciary marks were placed in the four corners of the image; these ensure that, as we delineate through the series, we are always on the same side aspect ratio and alignment. The T1w contrast of each voxel step was overlaid with 0.05 mm drafting film (Flat and Rotary Co., Ltd.) to permit superimposition of contrasts, where in conference with co-authors, the signatures of structures are identified and then drawn.

For some delineated structures, some apparently strong signals are still insufficient to fully delineate. For example, the external globus pallidus (EGP), is marked by a the black positivity in T2w, which in isolation can appear to delineate the EGP unambiguously; however, on investigation of the corresponding DEC image, the green directional information of the internal capsule (ic) distinguishes the medial and dorsal edges of the EGP more clearly. Further, some of the delineations were impossible to make purely from the MRI contrasts, i.e., the basal nucleus (B), where the T2 contrast assists in identifying its ventral most boundary, but no other contrast can complete it, and we had to rely inferences from histology to draw the remaining boundaries. The cortex of this map is based on the comprehensive histological cortical maps from Mai et al. (2016a, b).

Once satisfied with each voxel step/slice, we move to the next, and repeat the process until the range is complete and then repeat it again for each orthogonal plane. Albeit, this is not done in complete serialisation, following a structure, or small set of structures, across orthogonal planes is more efficient and accurate. These tracings are then digitised using Adobe software (Adobe Inc., 2019 Adobe Suite). At this digitisation step, a final sweep through the diagrams is done to harmonize delineations from level to level.

## Results

**Fig. 1:**
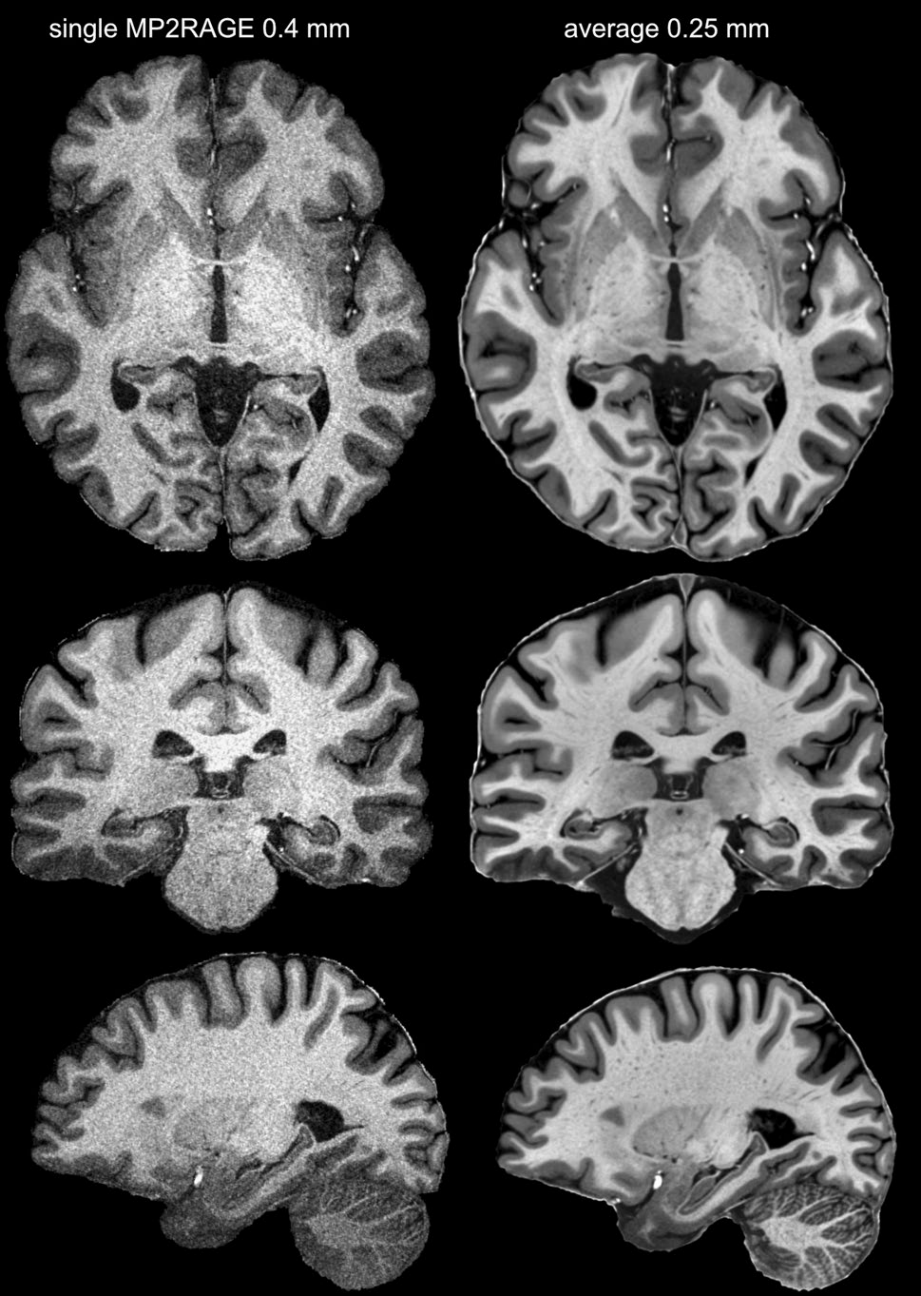
From single acquisition to high-resolution and quality average. The left panel shows the denoised and skull stripped (UNIDEN) image of a single MP2RAGE acquisition, while the right shows the average T1w image in 0.25 mm resolution. The left image shows considerable detail; this detail is masked by strong grain (noise). On the other hand, the images on the right are virtually noise-free and even small contrast variations can be relied on to reveal meaningful structural detail

Averaging multiple acquisitions through the ANTs multivariate template fitting resulted in significant quality improvement—from grainy high-noise single acquisition images to a satisfactory average within an individual subject (Fig. 1).

**Fig. 2:**
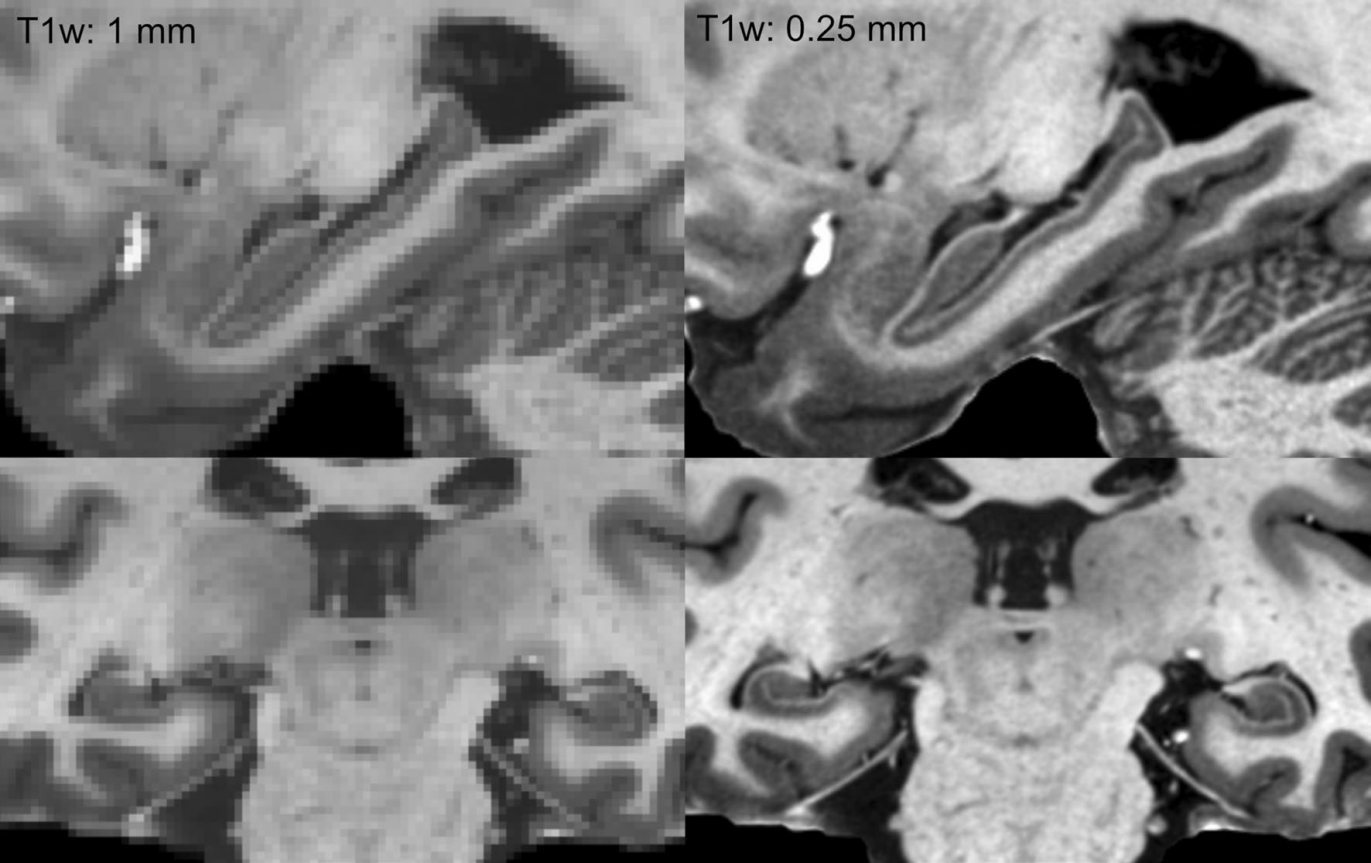
The importance of resolution in segmenting the hippocampus. The left column shows the dataset at 1 mm resolution, while the right column shows the exact same data at its original resolution of 0.25 mm

Our results also demonstrate fine detail in our final images, detail unavailable in the initial scans. They also highlight the importance of high resolution for imaging structural details, for example in the hippocampus (Fig. 2). Attempting to discern hippocampal subfields at 1 mm involves risky guessing (Wisse et al. 2021), even in excellent data as those in Fig. 2. In contrast, at 0.25 mm resolution, the subfields are clearly discernible in T1w images.

**Fig. 3:**
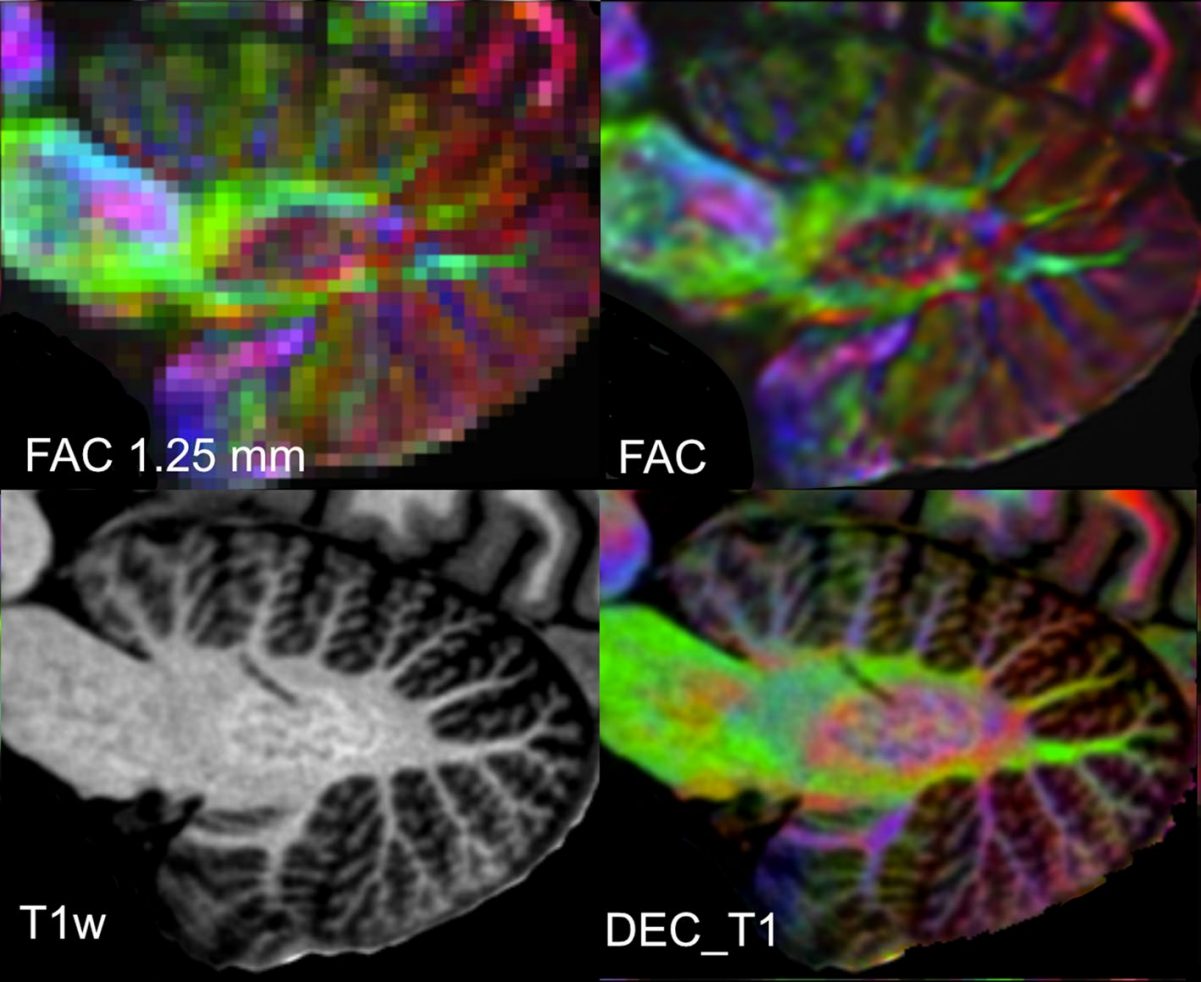
Sagittal plane showing the cerebellar dentate nucleus across a selection of contrasts. The first panel (top left) shows the FAC sampled at the scanning resolution of 1.25 mm. Note that the dentate can be clearly seen in both the T1w and the high-resolution FAC example, but most clearly in DEC_T1. This demonstrates the superior detail revealed in the right FAC image (red: LR axis or vice versa, blue IS axis, green AP axis, the same colour coding applies to all FAC or DEC_T1 images)

Our results also demonstrate the ability of post-processing to reveal structural detail finer than the acquisition resolution. Figure 3 shows the resolution gain achieved by the super-resolved processing for the FAC data. DWI was acquired at 1.25 mm isotropic; while this is a high-resolution for DWI, it is low compared to our T1w and T2w protocols. Comparing the acquisition resolution (top left) and with the high-resolution average in 0.5 mm^3^ (top right) demonstrates the detail that is achievable through our post-processing.

**Fig. 4:**
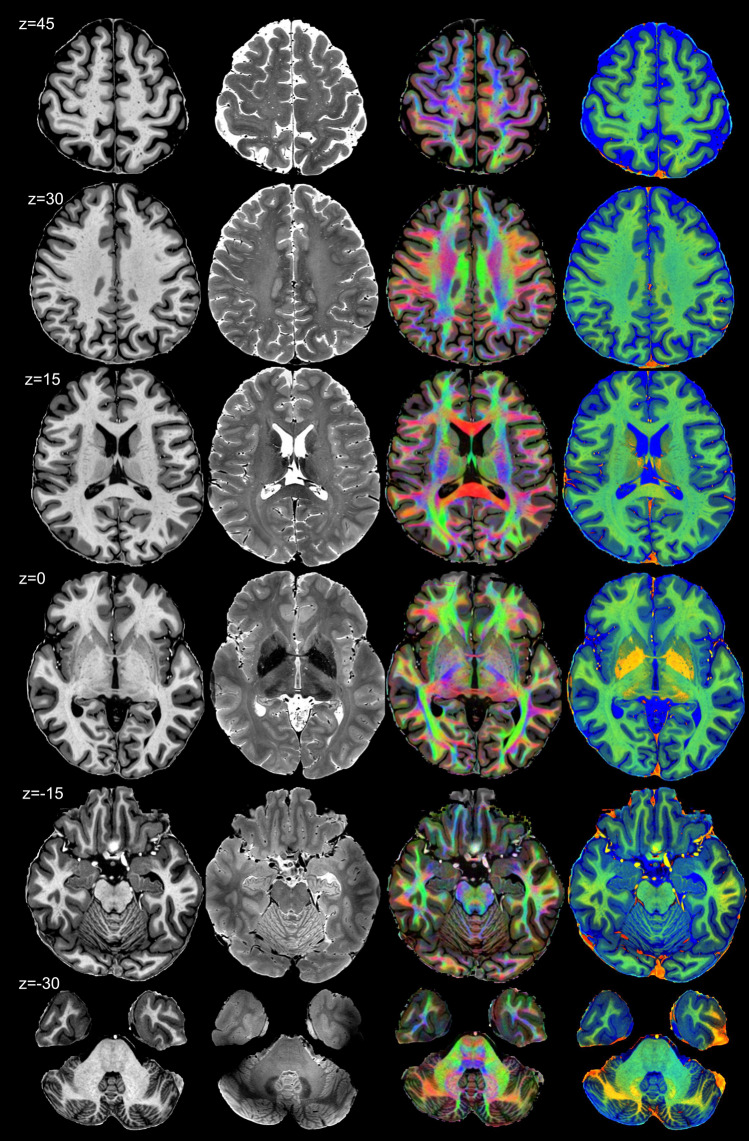
Horizontal sections from Subject 1. Each column displays a different contrast: T1w, T2w, DEC_T1, and ColorT1T2, and for each row, the z position relative to the AC-PC plane is labelled on the left. Note the excellent contrast, structure resolution, and sharpness

**Fig. 5:**
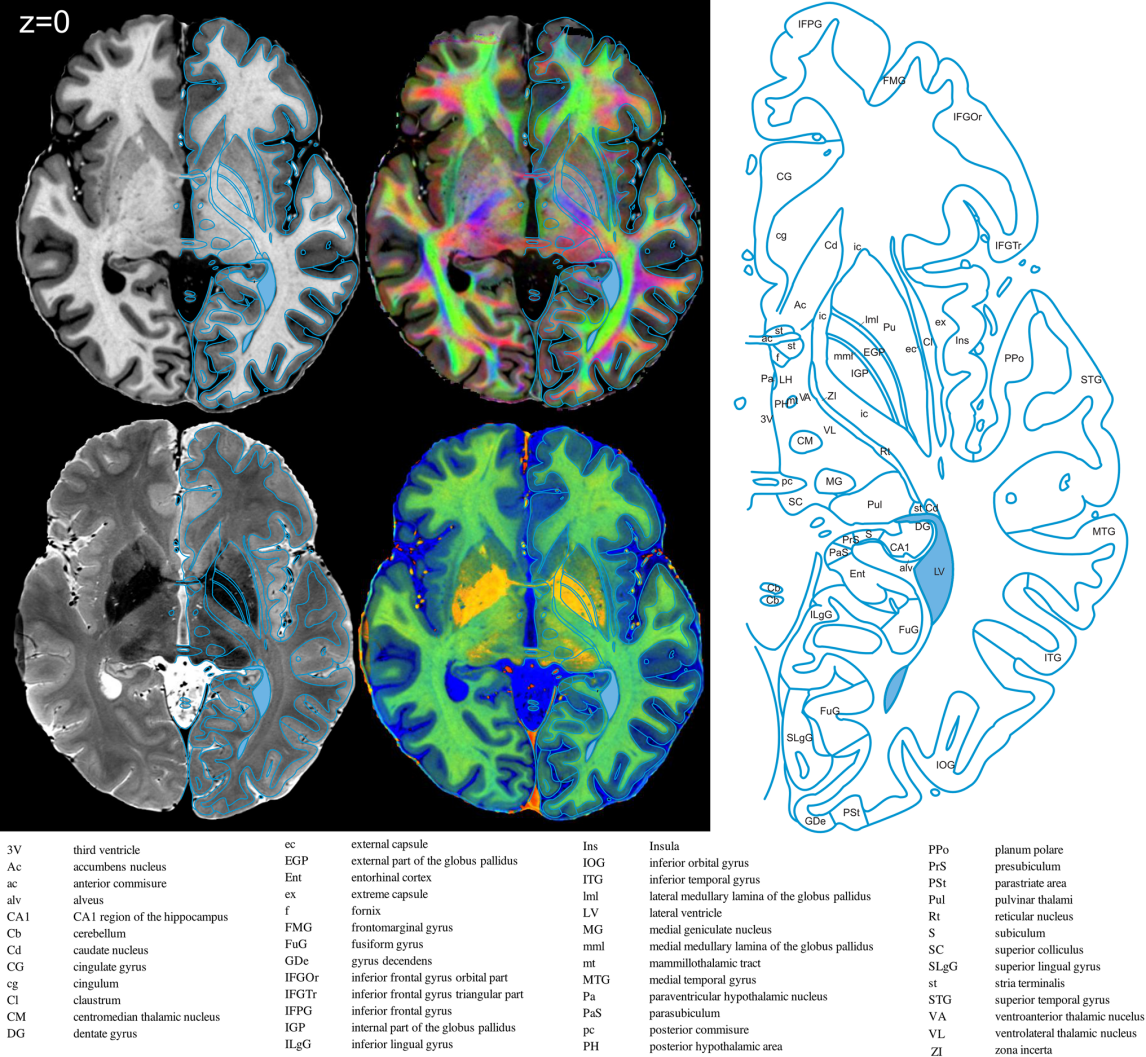
Atlas example from an axial (horizontal) section through the AC–PC line. Fifty three structures are identified, but we would like to point out that colours in the FAC suggest that additional subdivisions are possible, not currently undertaken

Having demonstrated that our analysis pipeline improves the available resolution, the next relevant question is whether the structural information revealed is sufficient to support accurate and comprehensive delineations of brain structures. Figure 4 displays a set of axial sections through the dataset. Figure 5 focuses on the axial plane through the AC–PC line (*z* = 0) on which we delineate 52 structures, for example the substructures of the globus pallidus and its flanking structures the putamen and the internal capsule. Visible are the thalamic substructures such as the ventral anterior nucleus, lateral thalamic nuclei, the medial geniculate nucleus, and the pulvinar. Posterior to the thalamus can be seen the hippocampal sub-regions—dentate gyrus, CA1, subiculum, and pre- and parasubiculum. A detailed segmentation of a coronal section of the hippocampus can be seen in Supplementary Fig. S3.

**Fig. 6:**
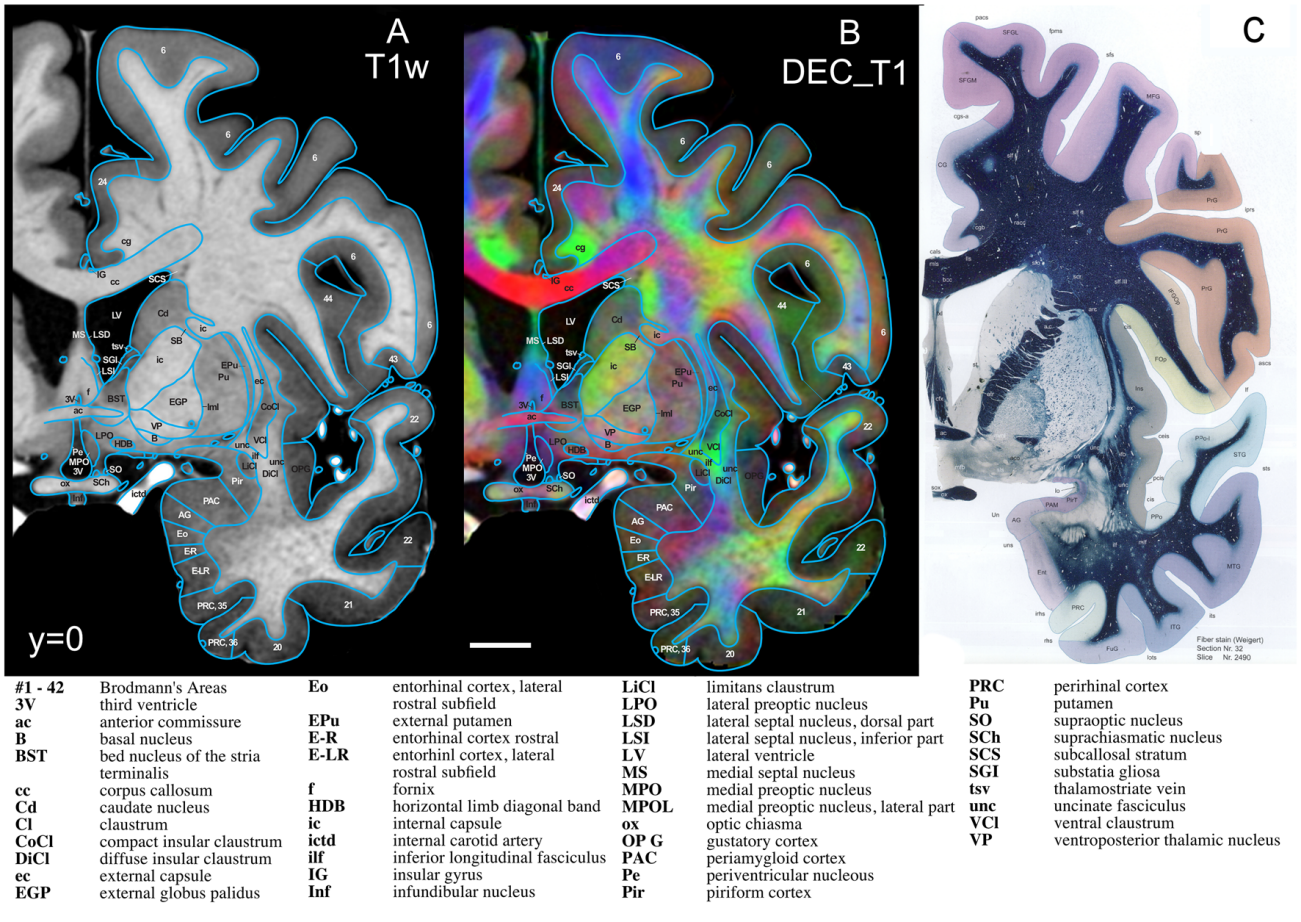
Coronal slices at the level of the anterior commissure (*y* = 0). **A** The T1w contrast and **B** the DEC_T1 contrast, both with delineations overlayed. **C** The corresponding section from Mai et al. (2016a, b) as reference for a comprehensive histology-derived atlas (C is reproduced with permission and is exempt from Creative Commons License). Also see Fig. 7 showing a zoomed in section justifying the delineations

Finally, we asked how such MRI delineations compare to delineations in the histology-based atlas of Mai et al. (2016a, b). Figure 6 shows a comparison at the level of the anterior commissure, demonstrating a level of delineations comparable to the gold standard documents.

**Fig. 7:**
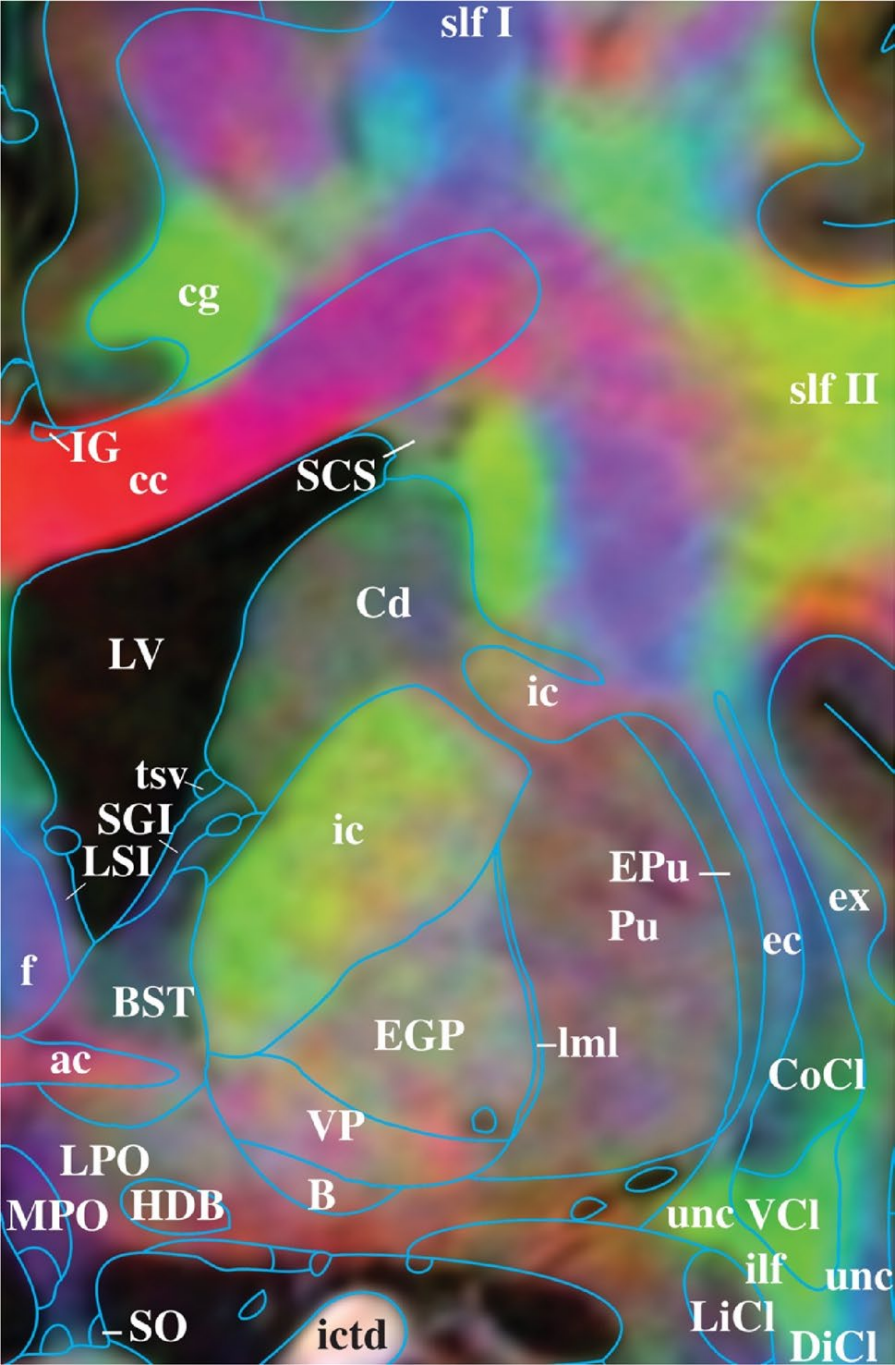
Zoom in on **B** of Fig. 6, highlighting the detail and contrast information even at high magnification, supporting the delineations provided. Legend as in Fig. 6. See also Supplementary Figs. S3, S4, and S5 for different contrasts and lower resolutions

To demonstrate the basis for these delineations, Fig. 7 shows a ‘zoomed in’ section and we will discuss ten structures. Each one demonstrates a link between MRI and histology, anatomy, dissections, and/or function, presented as a sample of the scope of referencing material used throughout the rest of the atlas. Figure 7 only shows the facilitating power of the DEC_T1 contrast, albeit the other contrasts are also used in the identifications and are shown in Supl. Figs. S3, S4 and S5. (1) The internal carotid artery (ictd) is best delineated by its bright white appearance in T1w and near black appearance in T2w (Huk and Gademann 1984). Blood vessels, depending on flow, do not always appear with the same signature in T1 and T2, but they appear vivid red in the ColorT1T2 contrast, hence a good mnemonic and a good distinction from nerves. (2) The corpus callosum (cc) is best identified by its fibre direction, predominantly appearing as vivid red, betraying the mediolateral direction of fibres, with brushes of blue laterally (Shah et al. 2021). (3) The cingulate bundle (cg) is identified in Fig. 7 by the vivid-green colour, signifying an anterior–posterior direction of fibres in the DEC_T1, but it also stands out as a darker grey in the T2w. (4) The superior longitudinal fasciculus, dorsal (slf I) and (5) superior longitudinal fasciculus, central (slf II), are both rather difficult to identify using only histology or T1w and T2w, but in the DEC_T1 contrast, thanks to directional information, the superior longitudinal fasciculus bundles are identifiable. Directional information reveals their borders where blue identifies a dorsal–ventral direction of fibres, into the superior frontal gyrus (slf I) and green, anterior–posterior (slf II), into the medial frontal gyrus (Janelle et al. 2022). (6) The inferior longitudinal fasciculus (ilf) is similarly difficult to identify on low-resolution FAC contrasts and even via myelin stains; however, in the DEC_T1 contrast, it is seen as a vivid-green. This colour accurately reflects the direction of fibres in the inferior longitudinal fasciculus anterior–posterior and is further validated by functional studies (Herbet et al. 2018). The inferior longitudinal fasciculus is also delimited by its separation from the limitans claustrum (LiCl) and ventral claustrum (VCl). (7) The uncinate fasciculus (unc) also presents as vivid green on the medial portion of and blue on its lateral. This correlates with its direction of fibres at AC =  + 0. The uncinate fasciculus is distinguished form the inferior longitudinal fasciculus, by the subtle shift to a darker grey in T1w and a subtle shift to brighter in the T2w, while the fibre orientation is identical to its neighbour the VCL (Bhatia et al. 2017). (8) The external globus pallidus (EGP) is recognisable by its signature dark black in T2w (Zhang et al. 2007), but also visualised by its mottled appearance in the DEC_T1, distinguishable from its surrounding structures. (9) The compart insular claustrum (CoCl) is separated from the ventral claustrum (VCl) by a colour shift from turquoise-green to lime-green, corroborated by hodological and histology studies of the area (Watson et al. 2017). Finally, (10) the fornix (*f*) is a structure less consistent in its directionality as it travels from the midline of the brain underneath the corpus callosum to the hypothalamus, here as it is specifically the ‘columns of the fornix’, it is identified by its dorsoventral fibres in blue at the midline.

It is the combination of histological, dissectional, and functional knowledge, as well as the information granted by the MRI contrasts that permit us to delineate the structures of the striatum and thalamus in Fig. 7. In other words, it is not purely the MRI contrasts that guide the delineations of structures in this atlas, but an explicit linking between histology and MRI generating a near-post-mortem detail of the living human brain. The features described in these ten anatomical examples from the HBA illustrate a logic that is applicable to the rest of the structures of the brain. Other human brain atlases offer different features complimenting and informing the present work (Ding et al. 2017; Hawrylycz et al. 2012; Sjostedt et al. 2015, 2020; Sunkin et al. 2013).

The final point to note that homology is the key in determining these ten labels, as well as almost every other structures identified throughout the brain. The human brain is almost entirely homologous across mammals and to some degree birds. Homologies, a structure being the same on one animal as it is in another, permit comparative anatomy between species. Homologies assist scientists to construct animal models of disease to test hypotheses that are inspired by human considerations on experimental animals and then related back to the human. As a result, we have lent on other animal brain atlases to inform our delineations. In particular, primate brains show virtually no structure unique in one, or absent in another species, this holds true when comparing human to marmoset monkey. With this in mind, the Atlas of the Marmoset, Rhesus (Hartig et al. 2021; Paxinos et al. 2009), Rat (Paxinos and Ashwell 2018; Paxinos et al. 2015, 2021; Paxinos and Watson 2014), Mouse (Franklin and Paxinos 2019; Paxinos et al. 2020), and even the Chick (Puelles et al. 2019), are all used to better inform structure delineation in the human brain.

## Discussion

Amongst the most important resources in neuroscience are atlases to navigate the brain. We have acquired magnetic resonance imaging data for the living human of quality that permits detailed segmentations. Provided here are these data (https://hba.neura.edu.au/data-sets and https://osf.io/ckh5t/) which will serve as the basis for an MRI atlas of the in vivo human brain, a dataset with sufficient resolution and contrast to support delineations rivalling histology-based atlases. This is shown in the detailed delineations from this dataset for a coronal and horizontal slice through the anterior commissure, with the DEC_T1 providing additional connectional information that are typically unavailable in histological sections. HumanBrainAtlas can meet current requirements of a modern atlas, offering the identification of structures in a format familiar to researchers and clinicians—in vivo MRI.

By averaging multiple low SNR images, we produced sharp virtually noise-free images (Shaw et al. 2019), without the need for additional in scanner hardware. This was achieved through sophisticated post-processing, increased availability of powerful computation hardware, and image analysis software, notably the ANTs, FSL, and MRtrix packages. Averaging multiple acquisitions, as employed in this project, is a viable approach to improve image quality and resolution. However, it is costly, and returns diminish with increasing numbers of acquisitions. Our work prioritised quality, opting for large numbers of repeats; while necessary, feasible, and desirable for this small number of subjects, one could argue that this is unfeasible for most applications. It would be worthwhile to determine what data quality could be achieved using a less-intensive measurement regime. As repeated measurements provide more and more diminishing returns, a smaller number of scans such as four or five repetitions, even relying exclusively on 3 T and slightly lower resolution would only result in modest losses of image quality. Presumably, acquiring quality MRI will become easier in the next 5 years, for example T1w and T2w at 0.5 mm or DWI data at 1 mm in reasonable scanning times. In the supplementary materials, we provide downsampled examples of Fig. 7 (Suppl. Fig. S3, S4 and S5), and this illustrates the combination of lower resolution MRI data, informed by high-resolution segmentation. The images, segmentations, and analysis scripts from the HumanBrainAtlas will facilitate the analysis and interpretation and of such future data.

Our DWI-derived images (FAC and DEC_T1) demonstrate that averaging repeated acquisitions in an up-sampled space can provide higher resolution than the native scanning resolution. The detail and quality of the FAC images at post-averaged resolution of 0.5 is noticeably superior (Fig. 3 top right) than the scanning resolution of 1.25 mm (Fig. 3, top left). This demonstrates that averaging multiple low-resolution images with small misalignments and distortions can be used to construct an average image with a resolution higher than that of the acquisition. Presumably, the efficacy of this approach rests on the number of images available for averaging. This super-resolution effect was most pronounced in the DWI data, because a ‘single’ DWI is an average of multiple images (33) and we averaged multiple DWI acquisitions (10). We suggest that a similarly strong effect could be achieved for functional MRI, which is also reliant on many repeated acquisitions, e.g., as demonstrated by Bollmann et al. (2017).

Resolution advantages of our T1w and T2w datasets resulting from our supersampled approach are much less obvious. The template resolution of 0.25 mm was chosen for several reasons, the two most important are (1) the small voxel size of the target space reduces the blurring that occurs from resampling a moved image. (2) A 0.25 mm template allows convenient and lossless sampling of discrete slices at 0.5 mm intervals for delineation. Our comparison of template reconstruction at 0.25 mm and 0.4 mm using identical parameters and inputs showed that the 0.25 mm template is sharper than the 0.4 mm template (Fig. S7). In their review on super-sampling, van Reeth et al. (2012) argue that new information can added by shifting an object in the scanner but only a small amount of information. Our observations confirm that for the T1w and T2w data, but we note that these benefits are additive. Difficult to say is whether the superior sharpness of our 0.25 mm over the 0.4 mm template is due to less blurring or super-resolution.

Techniques that do not damage the sample, such as in vivo MRI, offer advantages over histological approaches, specifically that the images and planes are aligned between contrast modalities. In histology, different stained sections are usually no better then 20 microns apart. Each histology section suffers from distortions unique to each. Histology, as any other post-mortem technique, also suffers from fixation shrinkage and warping. In the MR images provided here, each contrast is at every voxel step, with voxel steps analogous to tissue sections in this context, and with negligible distortions. Therefore, where histology requires new sections of tissue to compare the cytoarchitecture of the thalamus across different stains, MRI does not. Further, in histology comparing coronal and axial sections, it requires a new specimen—again, MRI does not. These fundamental advantages of MRI were previously insufficient to overcome the resolution disadvantage. Histological preparations still offer significantly higher resolution (< 1 micron); while the present resolution MRI is still only 250 microns; however, this no longer offsets the disadvantages for a whole brain atlas using in vivo MRI. Instead, the advantage of resolution in histology can only be leveraged in magnified views of small structures; as such, it is most suitable for research investigating neuroanatomy in small sub-regions of the brain.

Brain atlas templates are fundamental for neuroscience, being often the template in which other research is placed. Atlases can link theories of different fields. Readers assume that delineation were not constructed without input from theory, but with a comprehensive linking of literature and data. This allows the reader to understand more than just the anatomy for example the hippocampus, but also its molecular and cognitive relevance, and how it sits within a system. To link a molecular study in the mouse hippocampus region CA1 to a clinical study within the human, the most accurate and translatable definitions are required.

We argue that the dataset presented herein and made available for open access satisfies the needs outlined in the introduction: enabling a high-resolution atlas, free from tissue degradations (inevitable in post-mortem material) and in a contrast that is immediately familiar to the user of in vivo MRI. The forthcoming atlas, for which we present two sample pages here, but share more online, will be of value for researchers interested in human or animal nervous systems, clinicians interested in homologies or accurate interventions, and certainly educators. The field is indebted to histology atlases of human (Broadmann 1909; Büttner-Ennever et al. 2014; Economo and Koskinas 1925; Mai et al. 2016a, b; Nieuwenhuys et al. 1978; Talairach and Tournoux 1998), but we argue the future should rely on in vivo MRI.

### Supplementary Information

Below is the link to the electronic supplementary material.

Supplementary file1 (DOCX 5107 KB)

## Data Availability

The data presented as part of the manuscript are available under the Creative Commons Attribution-NonCommercial-ShareAlike 4.0 International License. The data and delineations are available at https://hba.neura.edu.au/data-sets/ and https://osf.io/ckh5t/.
