# Supplementary material for: HumanBrainAtlas: an in vivo MRI dataset for detailed segmentations
Source: Brain Struct Funct. 2023 Jun 5;228(8):1849–63. doi: 10.1007/s00429-023-02653-8 (PMC10516788; doi:10.1007/s00429-023-02653-8)
Supplement: Supplementary file 1 — Supplementary file1 (DOCX 5107 KB) [file 429_2023_2653_MOESM1_ESM.docx]

# Supplementary Materials

Table S1. Sequence parameter summary for Subject 1.

|  | Alias | Voxel Size | Dimensions | Usable Scans/Number of Scans Collected | TR/Ti (ms) | TE (ms) | FA (deg) | FOV (mm) |
| --- | --- | --- | --- | --- | --- | --- | --- | --- |
| T1w | Dutch | 0.50 x 0.50 x 0.50 | 312 x 468 x 500 | 6/6 | TR: 6000  Ti1: 1200  Ti2: 4790 | 3.18 | 8 & 9 | 250 x 234 x 156 |
|  | Flaws | Scans 1 to 3:  0.530 x 0.525 x 0.525  Scans 4 to 6:  0.570 x 0.5721 x 0.5721 | Scans 1 to 3:  256 x 374 x 400  Scans 4 to 6:  268 x 376 x 402 | 3/6  (scans 1-3 were discarded due to folding) | TR: 5000  Ti1: 620  Ti2: 1450 | 1.49 | 4 & 8 | 240 x225 x 192 |
|  | MP2RAGE | 0.400 x 0.4015 x 0.4015 | 396 x 528 x 528 | 12/12 | TR:4300  TI:700  TI2:2370 | 1.83 | 4 & 5 | 212 x 212 x 158.4 |
| T2 | Scans 1 to 4: | 0.33 x 0.3281 x 0.3281 | 384 x 640 x 640 | (scans 1 to 4, FOV to small) | 1330 | 118 |  | 212 x 212 x 140 |
|  | Scans 5 to 14 | 0.4 x 0.4 x 0.4 | 384 x 640 x 640 | 8/12 | 1330 | 118 |  | 256 x 256 x 154 |
| DWI |  | 1.25 x 1.25 x 1.25 | Scan 1,3-11:  192x192x118x33  Scan 2 192x192x36x33 | 10/11  Scan 2 was partial coverage aiming to optimize distortion but that failed. | 26.5s | 60 | 90 | 240 x 240 x 160 |

Table S2. Sequence parameter summary for Subject 2.

|  | Alias | Voxel Size [mm] | Dimensions | Usable Scans/Number of Scans Collected | TR [ms]  Ti | TE [ms] | FOV [mm] |
| --- | --- | --- | --- | --- | --- | --- | --- |
| T1w | Dutch | 0.50 x 0.50 x 0.50 | Scans 1 to 2:  320 x 468 x 500 | 5/5 | TR: 6000  Ti1: 1190  Ti2: 4790 | 3.16 | 160 x 234 x 250 |
|  |  |  | Scans 3 to 5:  312 x 468 x 500 |  | TR: 6000  Ti1: 1200  Ti2: 4790 | 3.18 | 156 x 234 x 250 |
|  | Flaws | Scans 1 to 4:  0.64 x 0.64 x 0.64 | Scans 1 to 3:  256 x 374 x 400 | 7/8  (scan 7 - file corrupted) | TR: 5000  Ti1: 620  Ti2: 1450 | 1.49 | 164 x 239 x 256 |
|  |  | Scans 5 to 8:  0.570 x 0.5721 x 0.5721 | Scans 4 to 6:  268 x 376 x 402 |  | TR: 5000  Ti1: 620  Ti2: 1450 | 1.49 | 145 x 203 x 217 |
|  | MP2RAGE | 0.400 x 0.4015 x 0.4015 | 396 x 528 x 528 | 12/12 | TR: 4300  TI: 700  TI2: 2370 | 1.83 | 158.4 x 212 x 212 |
|  |  |  |  |  |  |  |  |
| T2 |  | Scans 1 to 4:  0.4 x 0.4 x 0.4 | Scans 1 to 4:  320 x 512 x 512 | 8/12  (scans 1 to 4, folding) | 1330 | 118 | 128 x204 x 204 |
|  |  | Scans 5 to 11:  0.4 x 0.4 x 0.4 | Scans 5 to 8:  384 x 640 x 640 |  | 1330 | 118 | 153 x 256 x 256 |
| DWI |  | Scan 1  1.35 x 1.35 x 1.35 | Scan 1  180x180x104x24 | 1-5 | 22,500 | 84 | 243 x 243 x 140 |
|  |  | Scan 2  1.25 x 1.25 x 1.25 | Scan 2  192x192x130x24 |  | 38,000 | 74 | 240 x 240 x 162 |
|  |  | Scan 3-5  1.25 x 1.25 x 1.25 | Scan 3-5  192x192x128x33 |  | 32,000 | 68 | 240 x 240 x 160 |

| 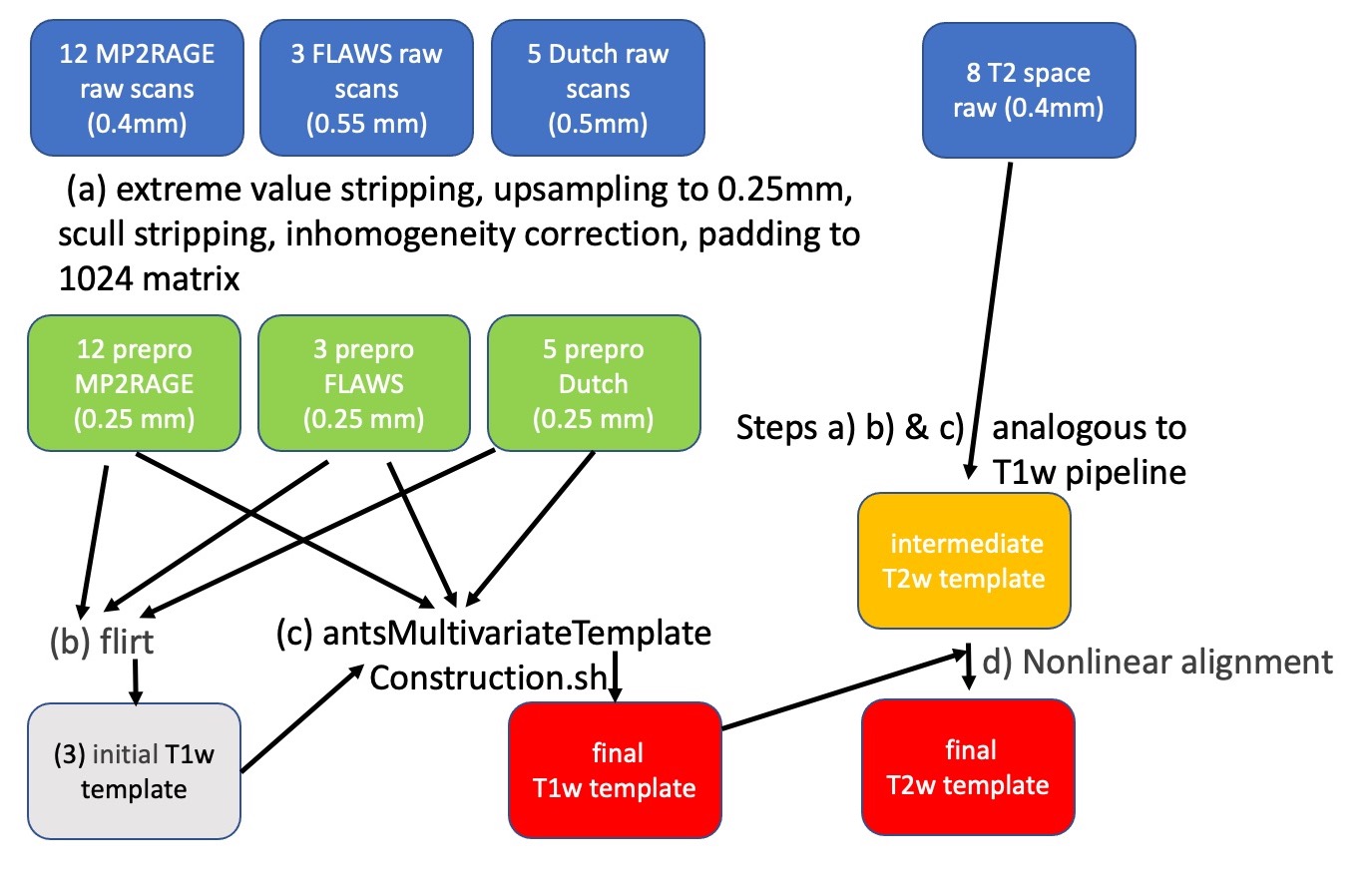 |
| --- |
| Fig. S1: Schematic of the processing pipleline for T1w and T2w data. T1w, firstly all datasets are subject to preprocessing, most of which does not involve any resampling (i.e. without blurring). Then from the preprocessed T1w (green) images the final T1w template is generated iteratively with the firs iteration generating a linearly averaged initial template. In the final step the antsMultivariateTemplateConstruction script constructs the final template warping the pre-processed T1w images guided by the initial T1w template to produce the final T1w template. The T2w is processed completely analogous but in an addition step the T2w template is aligned with T1w template. See methods section for additional details. |

| 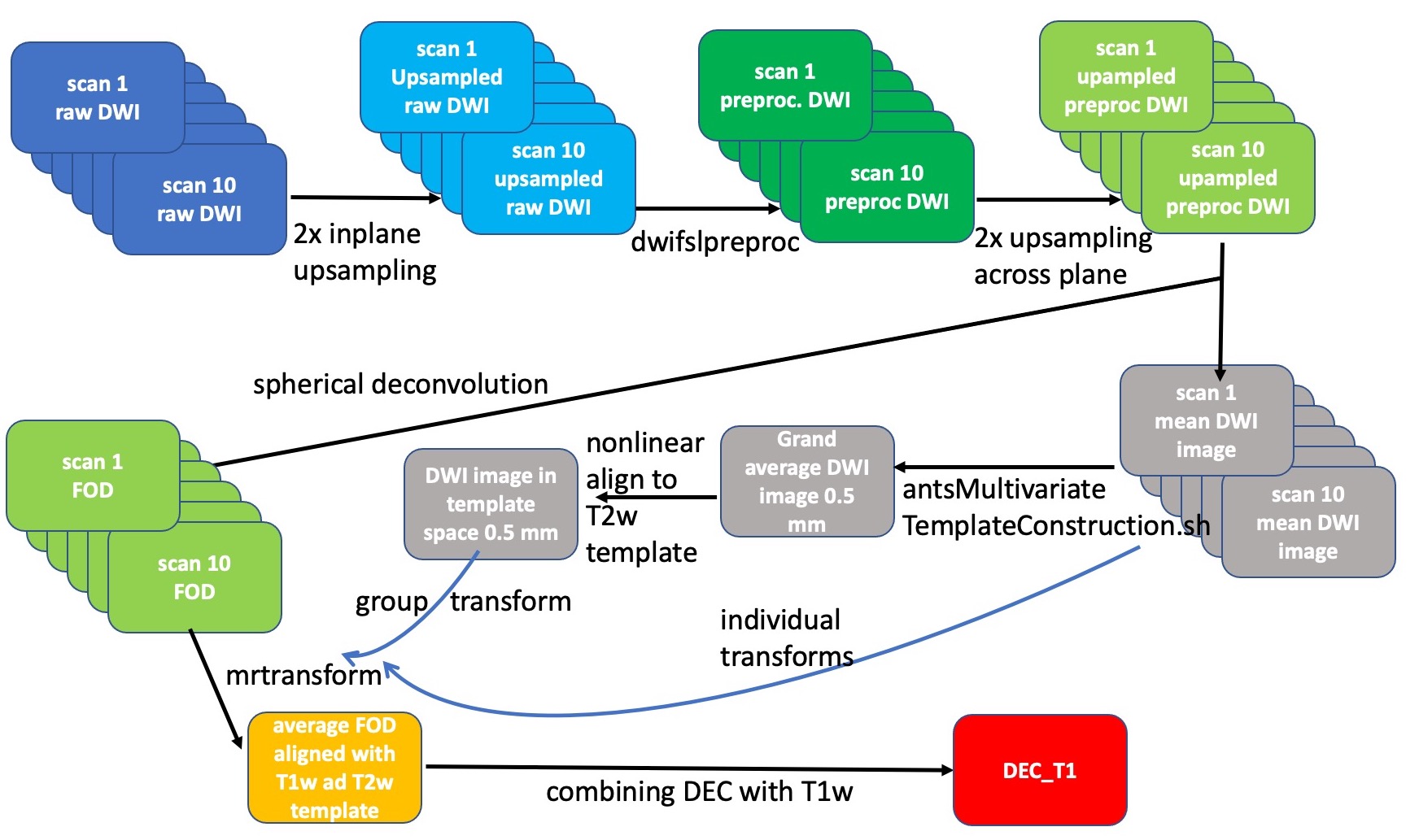 |
| --- |
| S2 DWI processing pipeline. First all 10 DWI sessions are independently pre-processed and up sampled. Then a mean DWI image is used to find estimate and alignment for each DWI session to the T2w template image via the antsMultivariateTemplateConstruction script. These transforms are then applied to the FODs from each session, thereby averaging them into the template space. See Methods section for additional details. |

| 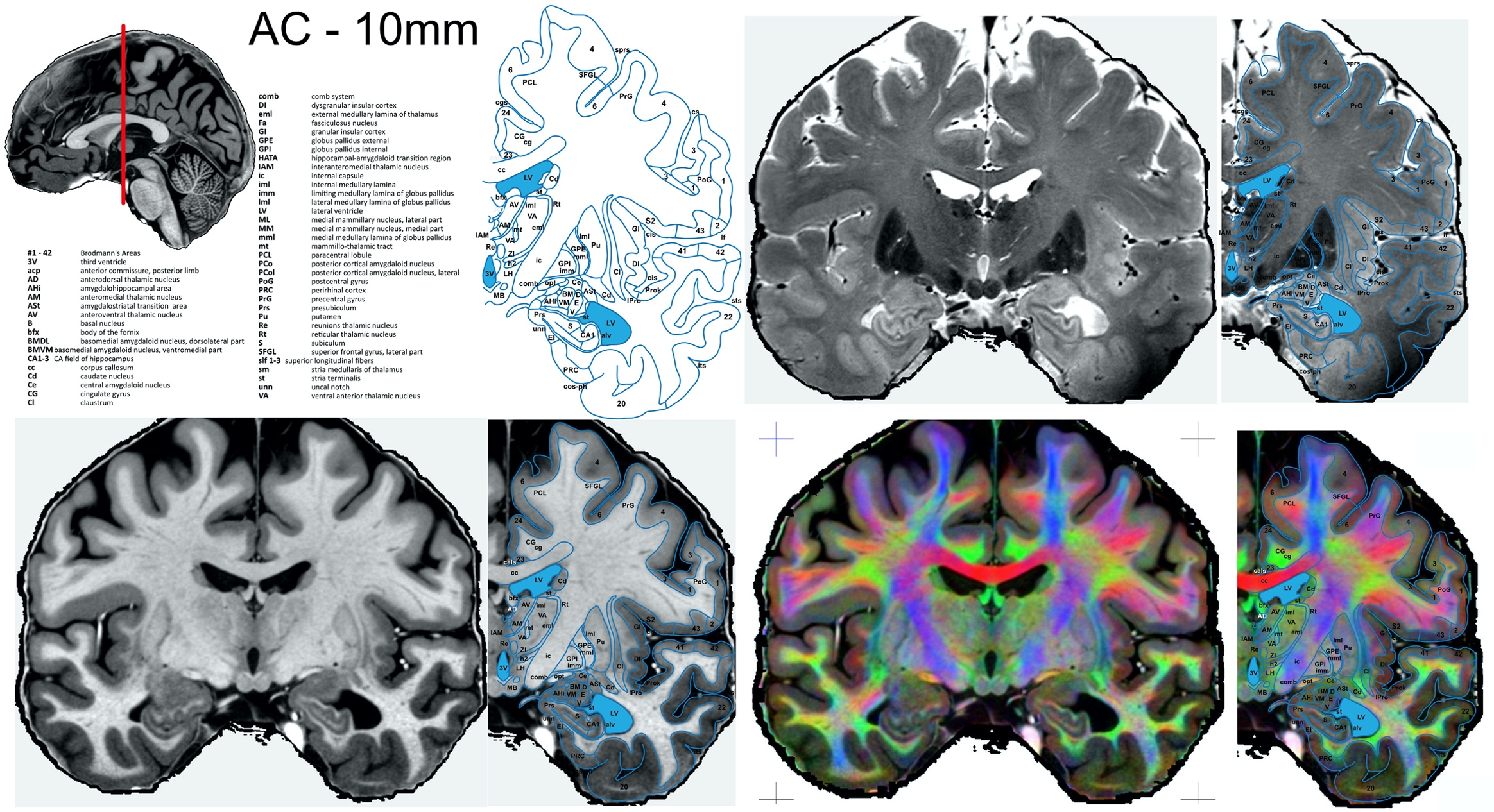 |
| --- |
| Fig. S1 Example atlas page at position AC – 10 mm, that is 10 mm posterior of the anterior commissure or y = -10. |

| 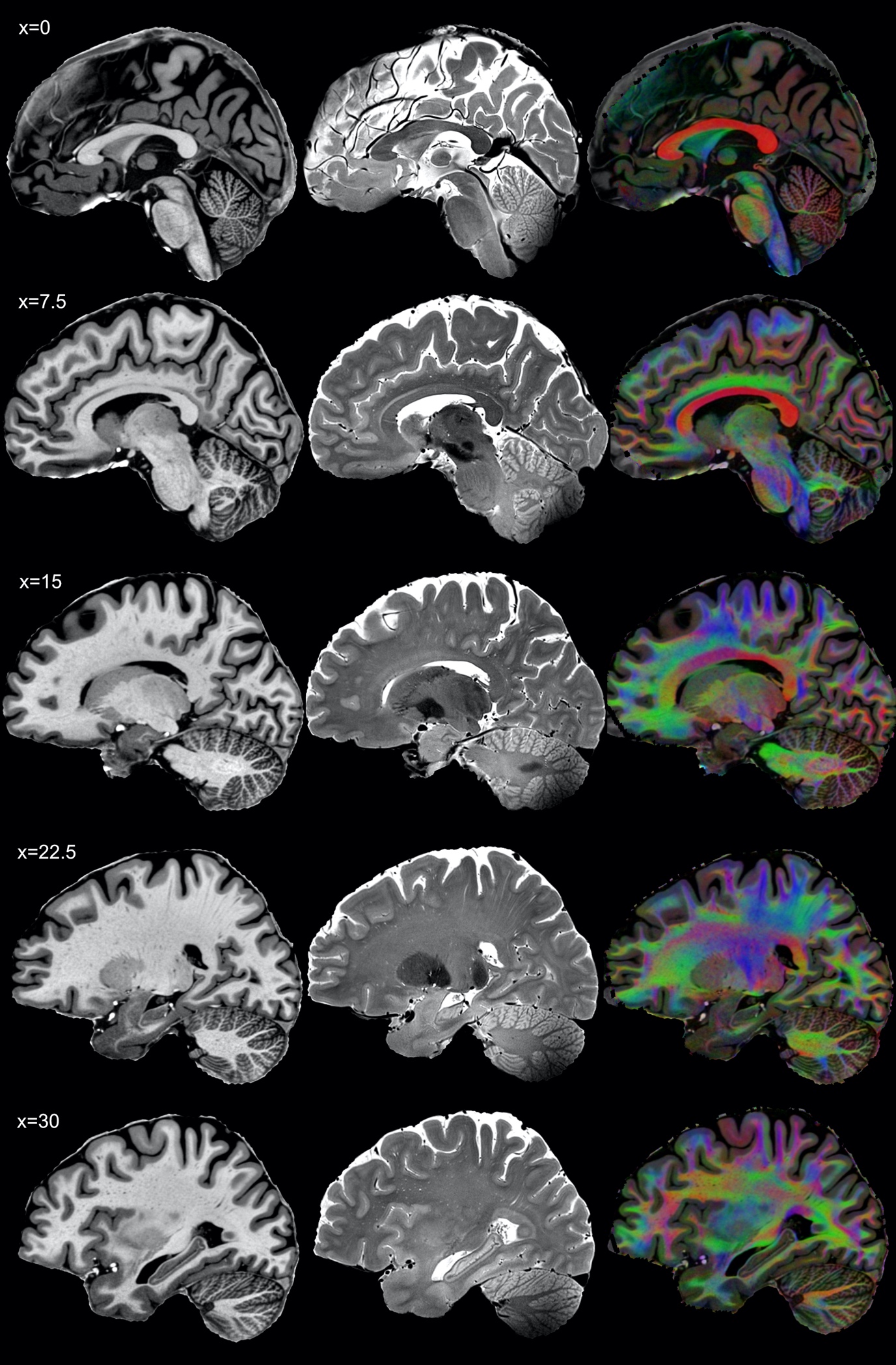 |
| --- |
| Fig. S2 Series of sagittal slices, complementing the horizontal and coronal sections presented in the main manuscript. |

| 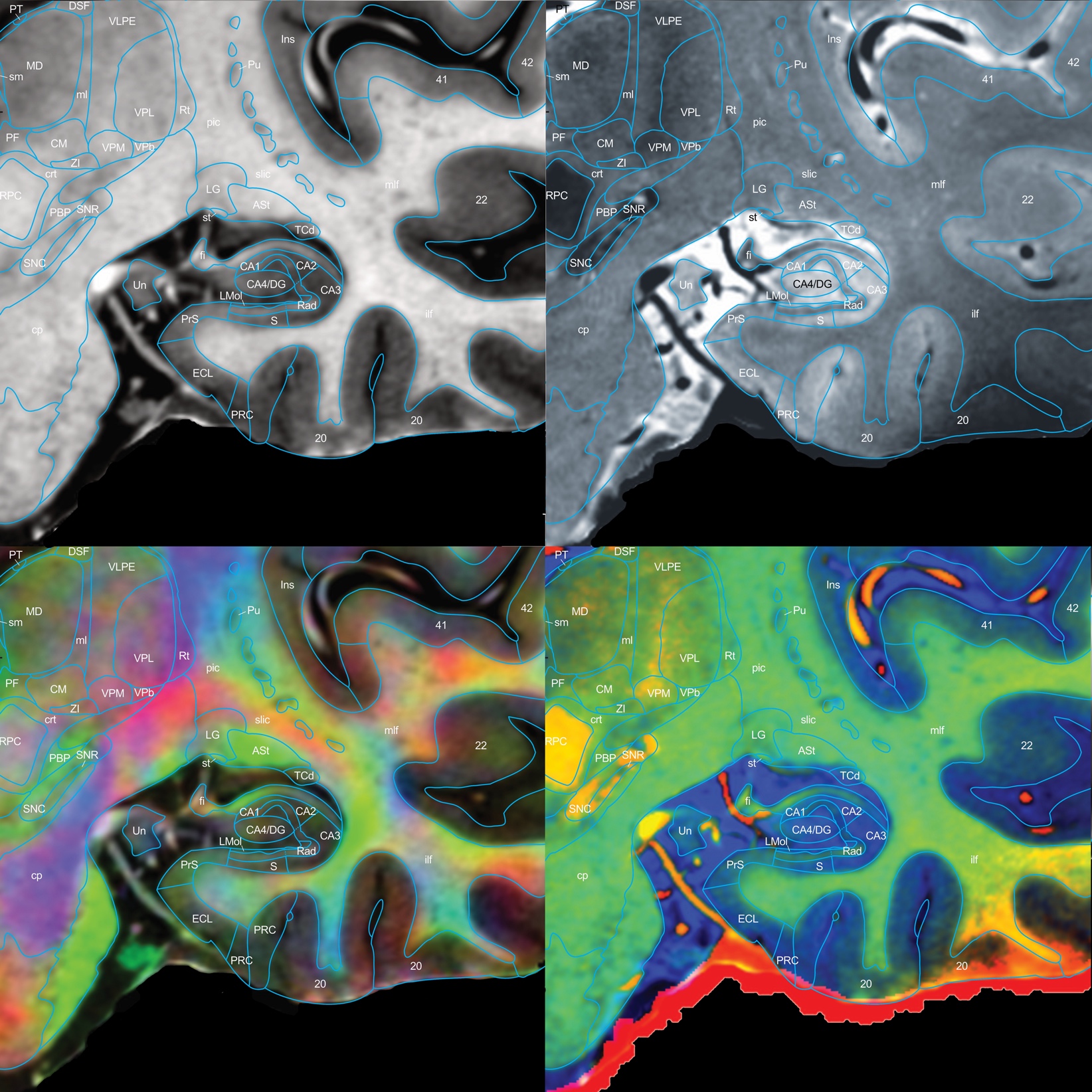 |
| --- |
| Fig S3. Detailed segmentation in the coronal plane, position AC -22.5mm, zoomed in on the hippocampus. The four contrasts used, T1, T2, DECT1, and T1T2, each offer unique markers to delineate the brain. You can follow the radium of the hippocampus (Rad) in black and green, in T2 and T1T2, respectively to generate its bounds, and by virtue of that the beginning of the subiculum (S), and dentate gyrus/CA4 (CA4/DG). We can also differentiate the fimbria (fi) from surrounding blood vessels (red in T1T2). The Uncus can be differentiated from surrounding blood vessels, nerves, and empty space in T1T2, T1 and FAC. The boundary of the cortical ribbon is clear on all contrasts (albeit with negligible differences). The shift in positivity in T1 and T2 contrasts delineates the shift from CA3 to CA2. The slightly positive layer in T1 above the Rad is identifiable as the lacunosum moleculare layer of the hippocampus (LMol). The differentiation of the cortical ribbon structures is done through inference from histology. With structures seen in the brain stem almost coloured-in across the four contrasts of differing positivity and colour.  PT = paratenial thalamic nucleus, DSF = dorsal superficial nucleus, MD = mediodorsal thalamic nucleus, sm = stria medularis of the thalamus, VLPE = posterior ventrolateral nucleus, external part, VPL = lateral thalamic ventroposterior nucleus, Rt = reticular thalamic nucleus, VPM = medial thalamic ventroposterior nucleus, VPb = basal ventroposterior nucleus, ZI = zona incerta, PBP = parabrachial pigmented nucleus, RPC = red nucleus, parvicelluar part, cp = cerebral peduncle, SNC = substantia nigra, pars compacta, SNR substantia nigra, pars reticulata, Pu = putamen, crt = cerebello-rubro thalamic fibres, PF = parafascicular thalamic nucleus, Ins = inusla, TCd = tail of the caudate nucleus, ASt = amygdalostriatal transition area, LG = lateral geniculate nucleus, PrS = presubiculum, ECL = entorhinal cortex, caudal limiting subfield, PRC(s) = perihinal cortex Brodmann’s 35 & 36, ilf = interior longtitudinal fascicle, mlf = medium longtitudinal fascicle, slic = sublenticular part of the internal capsule, pic = posterior limb of the internal capsule, st = stria terminalis |

| 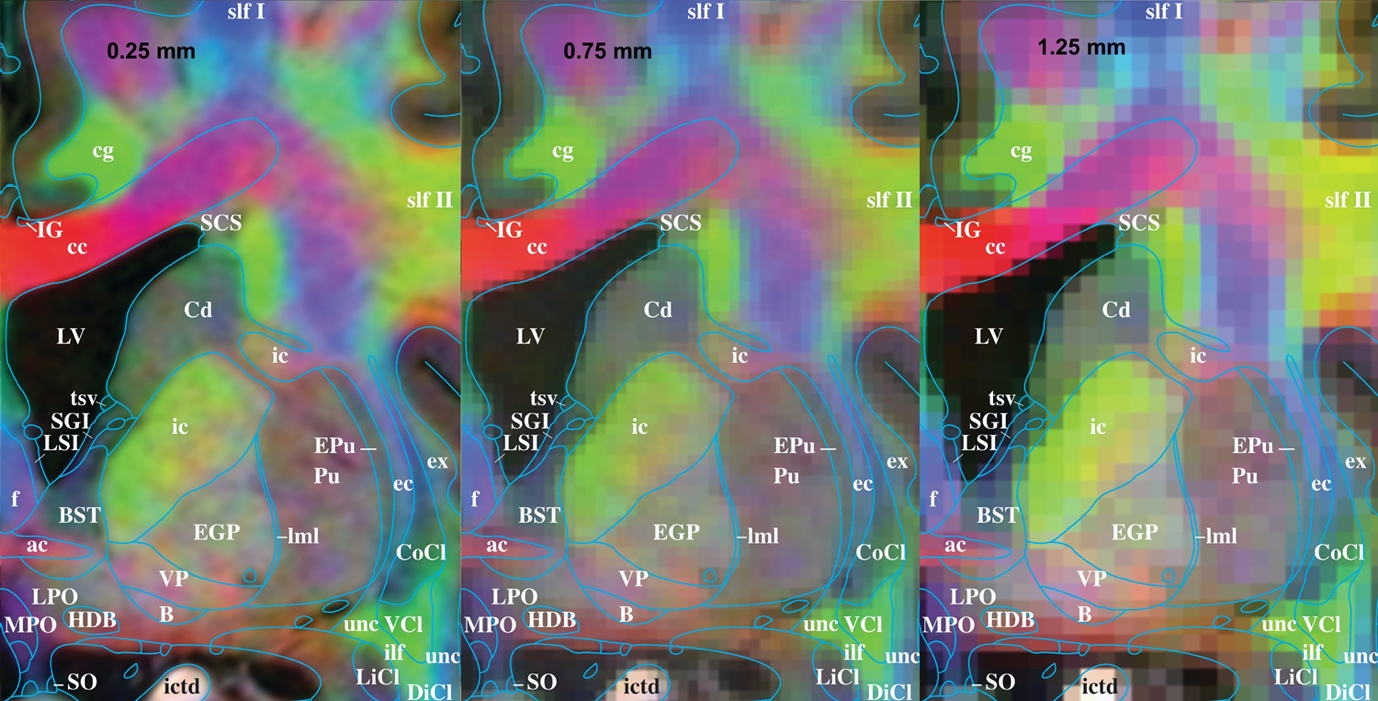 |
| --- |
| Fig S4. Comparing DEC_T1 images at 0.25 mm, 0.75 mm and 1.25 mm respectively. Despite clear loss of detail, the there is still significant structural detail at 0.75 and even 1.25mm. The interpretation of lower resolution images is significantly aided by the high-resolution example we provide. Our high-resolution data identifies the anatomical signatures, that allows the localisation of structures such as the bed nucleolus of the stria terminalis (BST), the internal capsule (ic), the external globus palidus (EGP) and the  pulvinar (Pu) which all show different fibre orientation profiles even in the low resolution  case. |

| 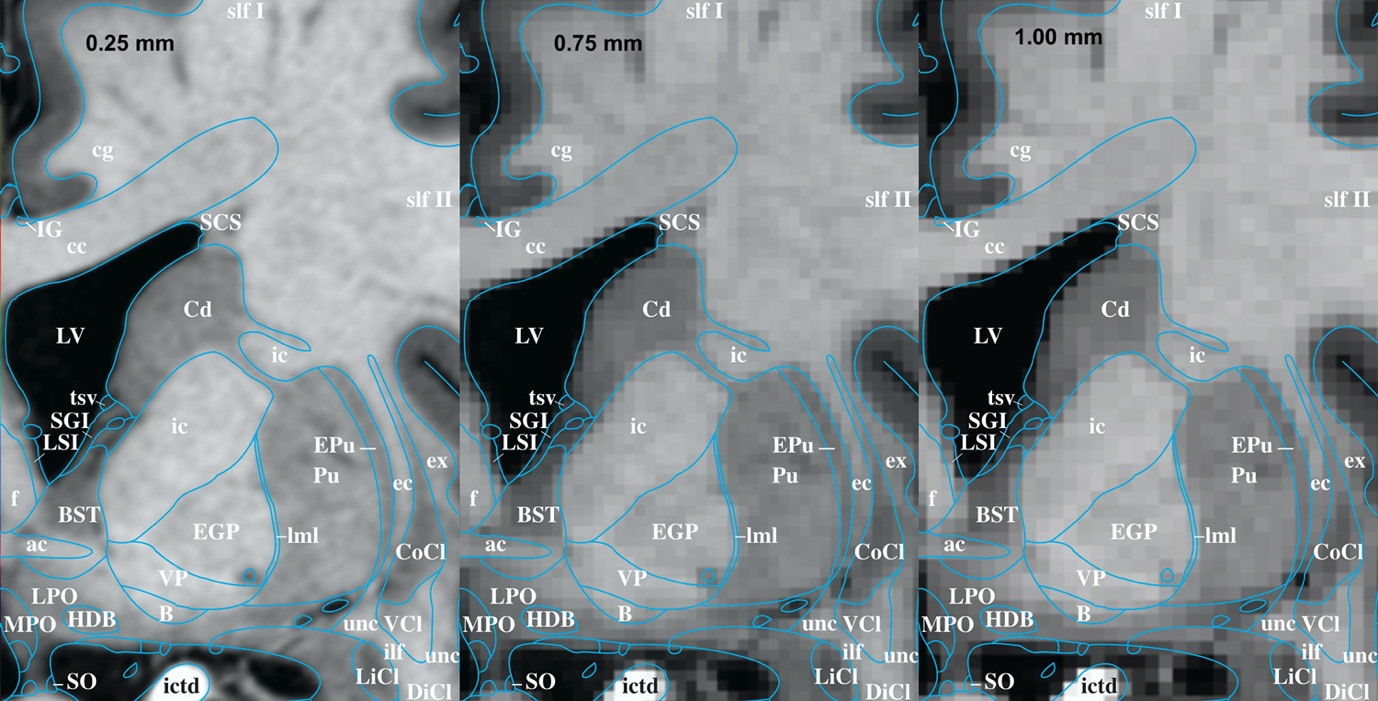 |
| --- |
| Fig. S5. Comparing T1w at 0.25 and 0.75 and 1 mm. These resolutions were chosen as 0.75 and 1mm are rather commonly chosen for high quality T1w images. |

| 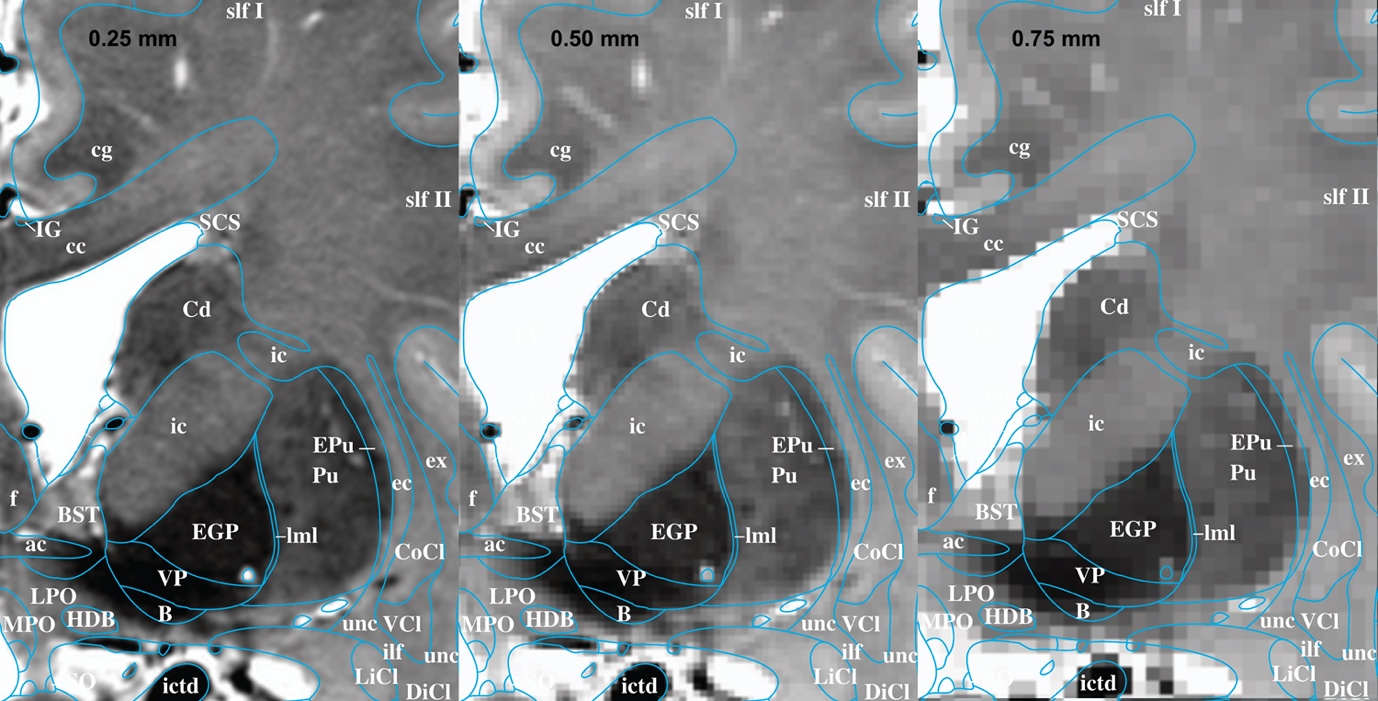 |
| --- |
| Fig. S6. Comparing T2w resolutions, here 0.25mm, 0.5mm and 0.75mm. Illustrating the loss of detail in T2w images. The high resolutions were chosen since such high resolutions are more practical for T2w than for T1w imaging sequences. |

| 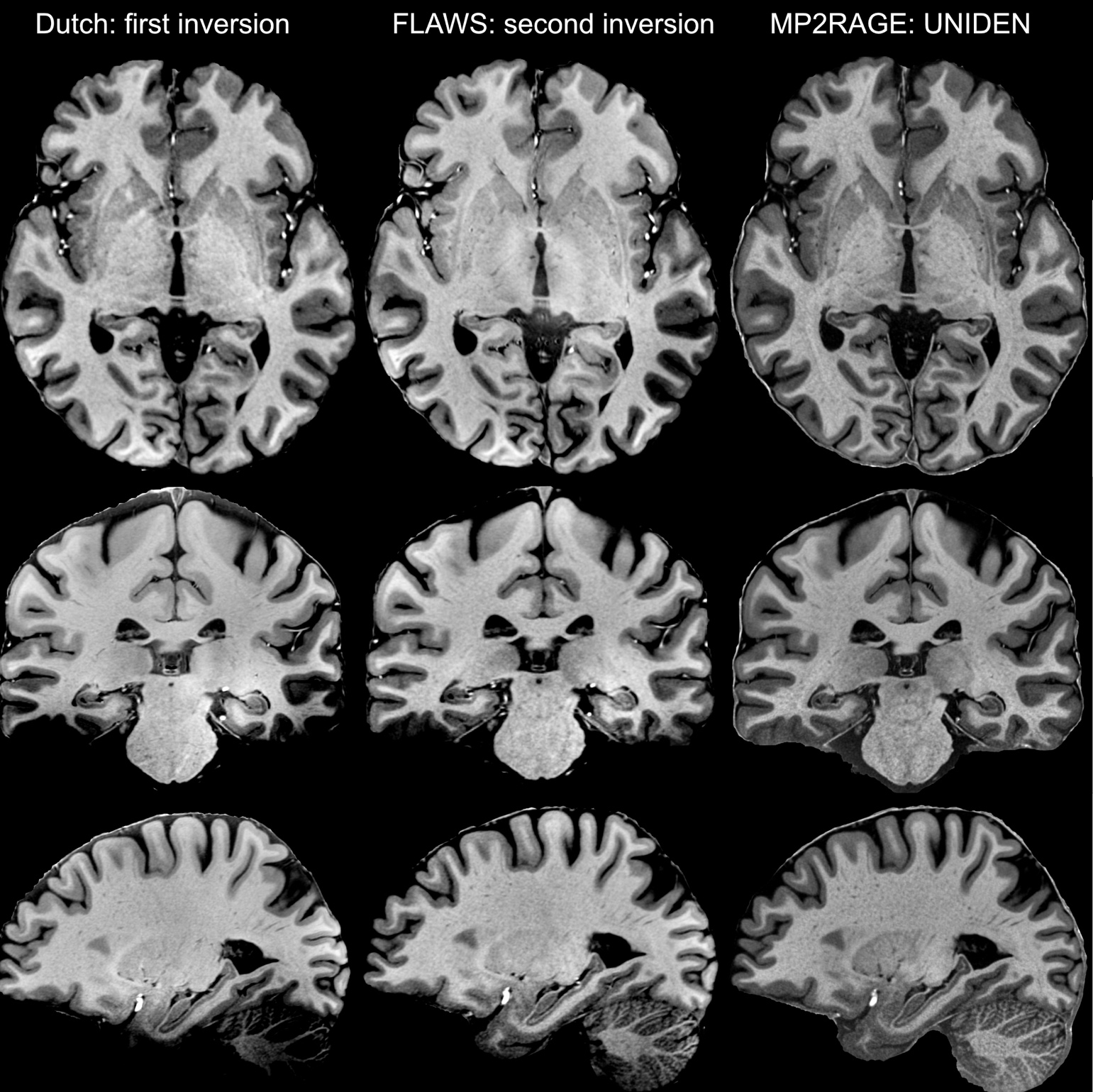 |
| --- |
| Fig. S7. Comparison of the different T1w protocols used. The **first row** shows an average of all datasets acquired with the ‘Dutch’ sequence, the **second row** shows an average image generated from all datasets acquired with the FLAWS sequence and the **last row** shows an average of images acquired with the MP2RAGE sequence. Judging with of the three T1w sequences would ultimately result in the best average was hard to judge from single acquisitions of each sequence. Single acquisitions of the FLAWS sequence appeared to show significantly superior contrast between subcortical structures where the MP2RAGE sequence appeared rather grainy. However after averaging multiple acquisitions of each, the MP2RAGE sequence resulted in better image quality than the other sequences. However, the other sequences were not useless, just less effective. Hence in the final average all three sequences are used. Note, here the non PD corrected version of the Dutch is shown, because the non PD corrected version is a little sharper. However it also has some in inhomogeneity in the cerebellum and the occipital pole. |

| 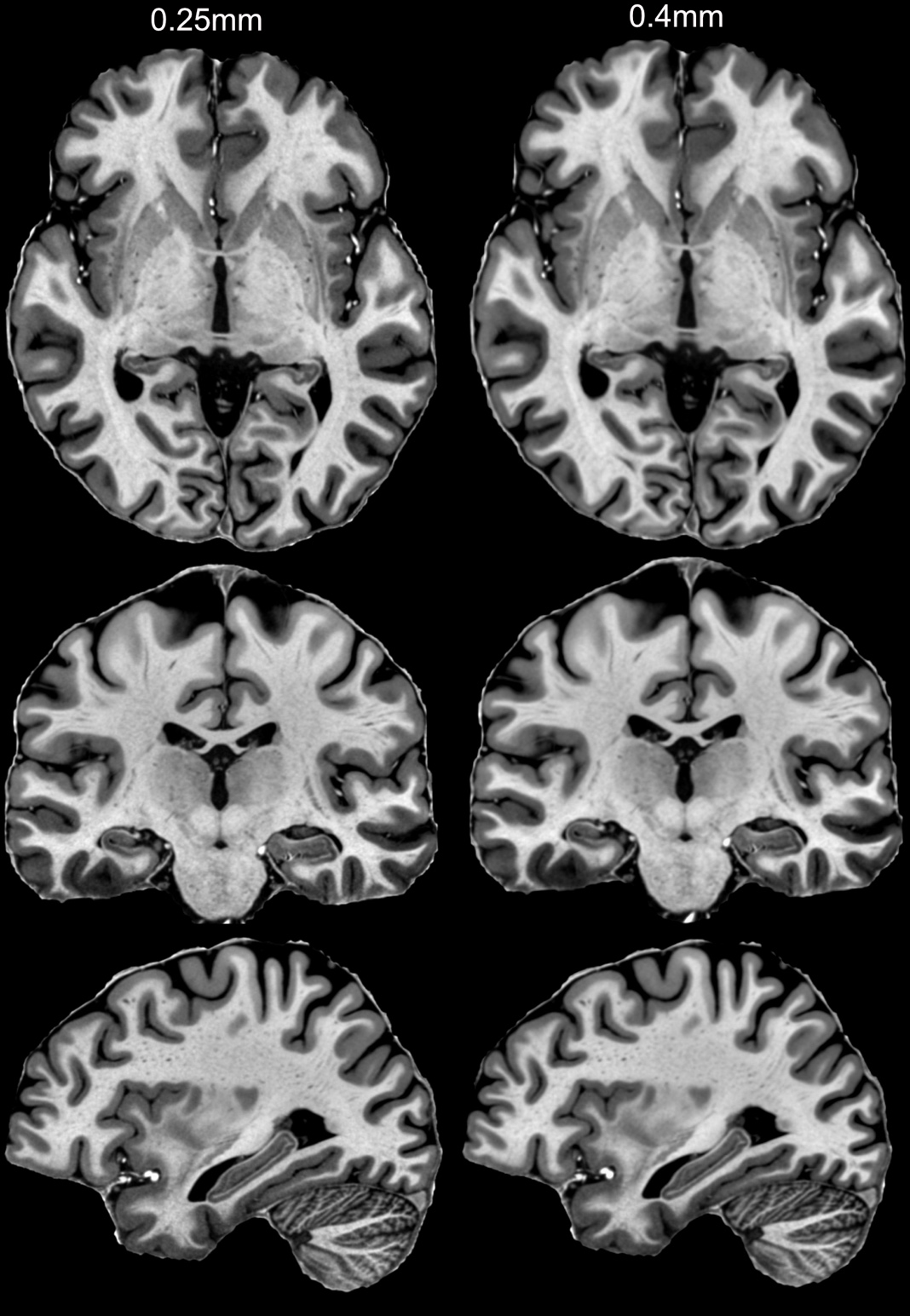 |
| --- |
| Fig. S8. T1w comparison at 0.25 mm and 0.4mm. The 0.4mm dataset was generated from scratch using another run of the antsMultivariateTemplateConstruction.sh scripts, but with a lower resolution target space. The lower resolution template is blurred compared to our 0.25mm template. |
